# Frequency, Mortality, and Predictors of Adverse Outcomes for Endocarditis in Patients with Congenital Heart Disease: Results of a Nationwide Analysis including 2512 Endocarditis Cases

**DOI:** 10.3390/jcm10215071

**Published:** 2021-10-29

**Authors:** Maarja Maser, Eva Freisinger, Leo Bronstein, Jeanette Köppe, Stefan Orwat, Gerrit Kaleschke, Helmut Baumgartner, Gerhard-Paul Diller, Astrid Lammers

**Affiliations:** 1 Department of Cardiology III-Adult Congenital and Valvular Heart Disease, University Hospital Muenster, 48149 Muenster, Germany; sefan.orwat@ukmuenster.de (S.O.); gerrit.kaleschke@ukmuenster.de (G.K.); helmut.baumgartner@ukmuenster.de (H.B.); gerhard.diller@ukmuenster.de (G.-P.D.); astrid.lammers@ukmuenster.de (A.L.); 2 Department of Cardiology I-Coronary and Peripheral Vascular Disease, Heart Failure, University Hospital Muenster, 48149 Muenster, Germany; eva.freisinger@ukmuenster.de; 3 Institute of Biostatistics and Clinical Research, University of Muenster, 48149 Muenster, Germany; leo.bronstein@ukmuenster.de (L.B.); jeanette.koeppe@ukmuenster.de (J.K.); 4 National Register for Congenital Heart Defects, Competence Network for Congenital Heart Defects, 13353 Berlin, Germany; 5 DZHK (German Centre for Cardiovascular Research), 13353 Berlin, Germany; 6 Department of Pediatric Cardiology, University Hospital Muenster, 48149 Muenster, Germany

**Keywords:** congenital heart disease, infective endocarditis, outcomes, mortality

## Abstract

Background: Infective endocarditis (IE) represents a major complication in patients with congenital heart disease (CHD) and is associated with high morbidity and mortality. The aim of this study was to analyse the frequency and outcome of IE in contemporary CHD patients based on all IE hospital admissions in Germany over a 10-year period. Methods: Based on data of all hospital admissions in Germany from 2009 to 2018, we identified all CHD cases with a diagnosis of IE. The data contained information on patient demographics, diagnoses, surgical procedures, and mortality. The primary endpoint of the study was endocarditis-associated mortality as well as major adverse events (defined as death or myocardial infarction, stroke, pulmonary embolism, sepsis, renal dialysis, resuscitation, or intubation). Results: Overall, 309,245 CHD inpatient cases were included in the analysis (underlying heart defects of simple complexity 55%, moderate complexity 23%, and complex heart defects 22%, respectively). Of those, 2512 (0.8% of all inpatient cases) were treated for IE. The mortality rate of IE inpatient cases was 6% with a major adverse events rate of 46%, and 41.5% of cases required surgical intervention. The overall IE associated mortality was lower in adult CHD cases compared to the 153,242 in adult IE cases without CHD (7.1% vs. 16.1%, *p* < 0.001). After adjustments using multivariable logistic regression analysis, the presence or complexity of CHD was not associated with the outcomes. Meanwhile, age, male sex, and co-morbidities emerged as significant predictors of adverse outcomes. Conclusions: IE accounts for a minority of CHD related hospitalizations but remains a deadly disease, and major adverse events are common in this setting. Due to different demographic and co-morbidity spectrums, adult CHD patients tend to have better survival prospects when compared to non-CHD IE patients. Acquired co-morbidities emerged as the main predictors of adverse outcomes.

## 1. Introduction

Infective endocarditis (IE) remains one of the major complications affecting patients with structural or valvular lesions, as well as those with congenital heart disease (CHD) [1,2,3]. With ongoing improvements in the early management of children born with CHD and better survival prospects of newborns with the condition, CHD has been transformed from a purely paediatric condition with a dismal prognosis, to a chronic life-long condition [3,4,5,6]. In the modern era, over 90% of children with CHD are expected to survive to adulthood in high resource countries [3]. However, most patients are not cured, and many are expected to present with residual or new valvular lesions during their life [3,4]. A considerable proportion of patients with CHD will also require valve related interventions or surgical procedures with prosthetic valves, which is also expected to increase the risk of IE further. Despite advances in medical and surgical management, mortality of IE has been reported as remaining high even in the current era and in experienced tertiary centres [7,8,9,10]. It has been suggested that IE accounts for approximately 4% of all admissions to tertiary centres [8], and the associated mortality is approximately 7% [9]. Depending on underlying CHD complexity or the presence of prosthetic material, individual mortality may be even higher, and CHD is perceived as a complicating factor in this setting [7]. More complex forms of CHD are often associated with heart failure, end-organ disease (e.g., renal disease), or multiple previous surgical or interventional procedures [1,3,5,8]. Therefore, avoidance and treatment of IE remains of major interest in the long-term care of CHD patients. However, it is unclear whether CHD or associated co-morbidities are the major drivers of adverse outcomes in this population. Furthermore, most previous research on IE in CHD has been based on data from individual expert centres with only limited information from large scale nationwide administrative datasets available.

Based on a dataset of all hospital admissions in Germany between 2009 and 2018, the aim of the current study was to assess the epidemiology of IE in CHD patients, clarify morbidity and mortality in this cohort, and identify risk factors for adverse outcome in this cohort. To clarify if CHD or associated conditions are major risk factors for adverse outcomes, the complexity of CHD and frequent co-morbidities were included as part of the comprehensive multivariate analyses, and IE in a large non-CHD cohort served as a control.

## 2. Methods

The study was performed based on the Nationwide German DeSTATIS Database (www.destatis.de, accessed on 1 July 2021). This database, maintained by the German Office for Statistics, covers all hospital admissions in Germany regardless of the patients’ insurance status. Registration of all hospital admissions is mandatory for all health care providers in Germany. As such, the dataset is complete regarding inpatient treatment in the country. In addition to patient demographics, all relevant ICD-10 codes covering the cause of admission with co-morbidities and associated conditions must be recorded, together with all operative and interventional procedures performed (using the German procedure specific OPS-code system). The dataset is hospitalization episode based and individuals are anonymized, therefore, no longitudinal patient follow-up and no detection of multiple hospital admissions of the same patient is possible.

Due to confidentiality reasons, data analysis was performed remotely using the DeSTATIS data warehouse. The analysis scripts were written using SAS software Version 9.4 (SAS Institute Inc., Cary, NC, USA) and submitted to the German Office for Statistics for further analysis. We identified all CHD inpatient cases with a diagnosis of IE in Germany throughout the 10-year period from 2009 to 2018. Complexity of CHD was classified as simple, moderate complexity, and complex CHD according to Bethesda disease complexity classification [11]. To account for the effect of age on occurrence and outcome of IE, patients were stratified into three age groups: children (<18 years of age), 18–40 years of age, and patients >40 years of age. The data contains information on patient demographics, diagnoses, surgical procedures, and mortality. Cases with missing information regarding sex or age were excluded from analysis. The primary endpoint of the study was endocarditis-associated mortality as well as major adverse events (defined as death or myocardial infarction, stroke, pulmonary embolism, sepsis, renal dialysis, resuscitation, or intubation). Diagnoses and procedures relevant to this study, including the respective ICD/OPS codes for CHD, are listed in Table 1.

The current study was performed in conformity with the Declaration of Helsinki and approved by the local Ethics committee as part of the umbrella research project using anonymized administrative health data (OptAHF project/2018-579-f-S).

### Statistical Analysis

Categorical variables are shown in absolute numbers and percentages, while continuous variables are given as mean ± standard deviation or median, respectively. Differences between groups were assessed by parametric tests or Chi-square tests, depending on data type. Clopper–Pearson confidence intervals were used for proportions. No adjustments for multiple comparisons were performed as part of the analysis. All analyses were intended to be purely exploratory (hypotheses generating), not confirmatory, and the results were interpreted accordingly. The association between co-morbidities and adverse outcomes was evaluated for two endpoints, death or MAE, using uni- and multivariable logistic regression analyses and odds rations (OR) where a 95% confidence interval (CI) was presented.

## 3. Results

**Figure 1:**
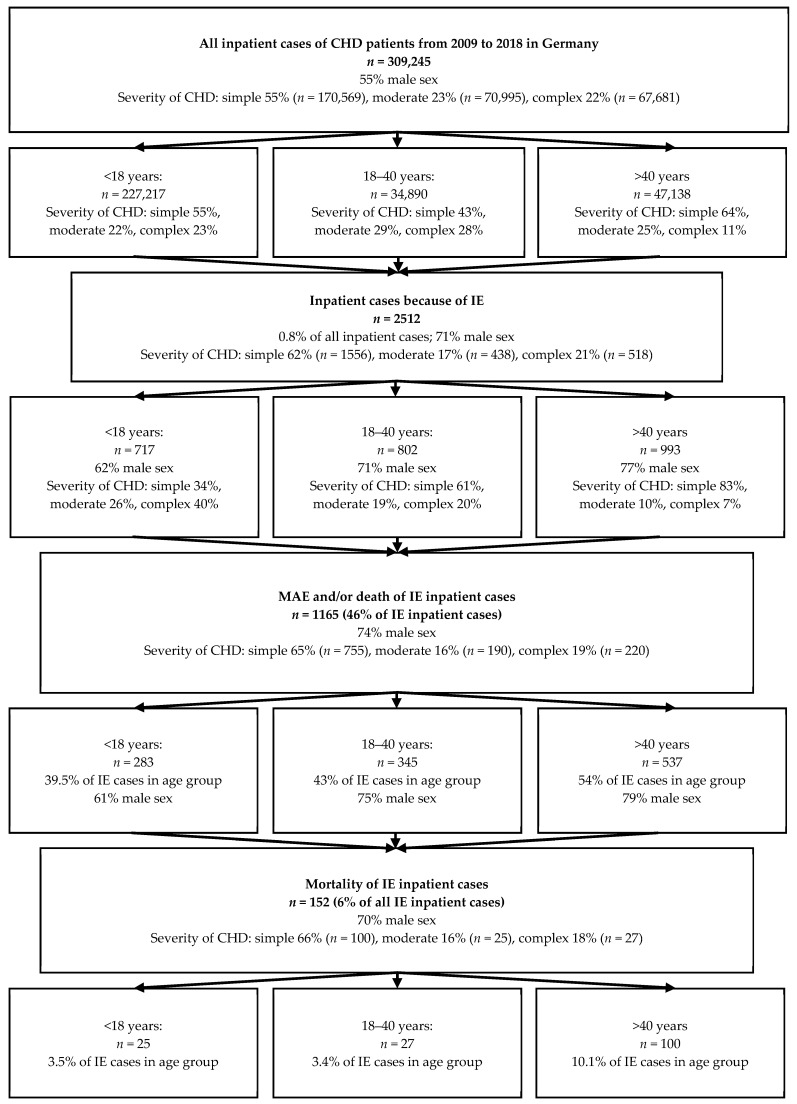
Frequency, Mortality and Major Adverse Outcomes for IE in CHD patients from 2009 to 2018 in Germany. CHD = congenital heart disease; IE = infective endocarditis; MAE = major adverse events.

The results of this study are illustrated in Figure 1. Over the 10-year study period 309,245 inpatient cases involving CHD patients were recorded. A total of 55% of all inpatient cases were male sex and the mean age at hospitalization was 14 years of age. The majority of cases, 55%, had simple underlying CHD conditions, while 23% of cases were of moderate complexity CHD, and 22% of cases were of high complexity CHD. Stratifying CHD cases by age at the time of hospital admission illustrated that most hospital admissions, 74%, occurred in childhood (*n* = 227,217; median age 2.3 years; 54% male sex). In addition, 11% of hospital admissions occurred in the age group between 18–40 years (*n* = 34,890; median age 28 years; 53% male sex), and 15% of admission were in CHD patients above the age of 40 years (*n* = 47,138 cases; median age 58 years; 59% male sex).

Focusing specifically on IE showed that IE accounted for 2512 inpatient cases in CHD patients (0.8% of all hospital admissions). Assessing IE cases by age group showed that the majority (*n* = 1795; 71.5%) of cases occurred in adult individuals, while 28.5% cases (*n* = 717) were seen in children. A male preponderance was seen in all age groups; 71% of all IE inpatient cases were of male sex. Stratifying the IE inpatient cases by CHD complexity showed that 62% of the inpatient cases had simple CHD lesions, 17% had moderate complexity lesions, and 21% had high complexity lesions. Complexity of CHD in IE cases differed between age groups: in paediatric population 40% of the inpatient IE cases had complex CHD, compared to 20% between 18 and 40 years of age, and only 7% over the age of 40 years.

Overall, the mortality of all the IE inpatient cases with CHD was 6% (*n* = 152). With that, IE accounted for only 2% of all deaths in the CHD inpatient cases (from 2009 to 2018, 7320 CHD patients died during hospital admission in Germany). In-hospital mortality was 3.5% in children (median age at death 2 years), 3.4% in cases 18–40 years of age (median age at death 31 years), and 10.1% in cases above 40 years of age (median age at death 61 years). Disease complexity was not directly related to case mortality. Fatalities totalled 6.4% of IE cases in simple lesion patients, 5.7% of IE cases in moderate complexity lesion patients, and 5.2% of cases in complex lesion patients. Similarly, on raw data analysis, case mortality was not different between male and female sex.

Assessing a composite endpoint of major adverse events and/or death (MAE, see Methods section for details) revealed MAE to be common in the setting of IE. Overall, 46% of IE cases (*n* = 1165) were associated with MAE. The occurrence of MAE was noted in 39.5% in paediatric population, 43% of patients between 18 and 40 years of age, and 54% of those over 40 years of age. No clear association between disease severity group and MAE was seen (48.5% of simple IE cases; 43.4% of moderate complexity cases, and 42.5% of complex CHD cases).

In total, 41.5% of IE cases in the CHD population (*n* = 1042) required cardiac surgical procedures during the same hospital stay. This included 18% of the paediatric cases (*n* = 128), 39% of patients between 18 and 40 years of age (*n* = 313), and 60.5% of IE cases over 40 years of age (*n* = 601). Among the 152 deceased patients, 59.2% (*n* = 90) had undergone a cardiac operation for IE. This ratio was 40% in paediatric population and increased to 70% in those over the age of 40 years.

In addition to CHD cases, 153,242 IE episodes in patients without CHD occurring in Germany over the study period were included. Many of these cases (*n* = 151,981; 99.2%) were in adult patients, with only 0.8% of cases occurring in children without CHD (*n* = 1261). Case mortality in these non-CHD IE cases was 2.1% in children and 16.1% in adult individuals (<18 years of age *n* = 27, >18 years of age *n* = 24,453).

To assess the association between CHD and patient demographics, or co-morbidities and outcomes, multivariable logistic regression analyses were performed across the spectrum of IE as well as specifically in IE cases with CHD. As illustrated in Table 2 and Table 3, CHD complexity in adult population was not associated with an increased risk of death in the overall cohort, or specifically in CHD patients. In contrast, co-morbidities such as heart failure, renal disease, or oncologic diagnoses were statistically associated with a higher risk of death in adult patients.

Similarly, in the paediatric population case mortality was not related to the presence or complexity of CHD, but rather co-morbidities were statistically associated with a higher risk of death (Table 4 and Table 5).

A similar picture emerged when major adverse events or death were considered. Again, both in the overall and in the CHD population the occurrence of MAEs was statistically associated with co-morbidities, but not with complexity of underlying CHD (Table 6 and Table 7).

## 4. Discussion

The current nationwide study covering over 300,000 hospital admissions in CHD patients over a 10-year period provides important insights into the occurrence of in-hospital mortality associated with IE in this population. While overall, IE was observed in 0.8% of all CHD hospitalizations and was only related to 2% of in-hospital deaths of CHD patients, IE remains a deadly disease with a case fatality rate of 6% across the age spectrum of CHD. However, stratifying patients by age at IE revealed that IE is both rare and less often deadly in paediatric populations (0.3% of hospital admissions in this age group were IE related, with a case fatality rate of 3.5%), especially compared to patients over the age of 40 (2.1% of hospital admissions in this age group IE related, with a mortality rate of 10.1%). In addition, major adverse events were common across all age groups, and approximately 40% of IE cases required cardiac surgical interventions. When assessing risk factors for adverse outcomes in comparison to non-congenital IE cases, we found that a diagnosis of CHD or complexity of CHD were not directly associated with adverse outcomes. Rather, we found that acquired co-morbidities such as diabetes, a diagnosis of heart failure, occurrence of renal dysfunction, coronary heart disease, or pulmonary disease were significantly associated with adverse outcomes. This interpretation is supported by the fact that mortality of IE in adult CHD inpatient cases (who are likely to be younger and have less co-morbidities) was lower compared to adult non-CHD patients (7.1% vs. 16.1%).

The overall IE mortality rate in the current study (6.0%) is comparable to a previous study including 164 IE episodes from a tertiary centre reporting an IE related mortality of 6.9% and slightly lower compared to a Dutch registry focusing specifically on CHD patients with prosthetic valves [7,9]. Similarly, the frequency of surgical interventions (41.5%) in our study was comparable to that reported by Tutarel et al. [9] at a large UK based adult CHD centre (37.2%). The current study adds to the literature by including a much higher number of affected inpatient cases and by relating IE frequency and outcome to different patient age groups and comparing these results directly to those seen in IE individuals without CHD. The major finding in this analysis was that demographic factors, such as increasing patient age in adult individuals and acquired co-morbidities appear to be more important determinants of adverse outcomes than the presence or complexity of CHD. This finding has direct clinical implications as it suggests that treating physicians should look beyond the obvious underlying CHD anatomy and focus on physiological factors and end-organ disease in these vulnerable patients. This view is supported by the notion that CHD patients did not have higher case mortality rates compared to non-CHD IE patients after accounting for relevant co-morbidities. Consistent with previous studies, the mortality rate of IE in adults with CHD in our analysis was lower compared to general non-congenital cohorts [12]. The reasons for this observed lower mortality in adult CHD patients remains speculative. Beyond the generally lower patient age at presentation, many endocarditic lesions in this cohort are right heart-sided [1,8,9,13]. Furthermore, therapy tends to be concentrated at expert centres and this may positively affect the outcome, as this generally applies for adult CHD patients [6,14]. It has also been argued that being confronted with the potential risk of IE since early childhood creates awareness of the possibility of developing IE in patients and primary treating physicians. This might lead to a timely diagnosis and proactive treatment in this cohort [9]. This also highlights the importance of adequate patient information, partnering with affected individuals and their parents, and educating them as well as physicians involved with the primary care for this patient group [9,15,16,17,18,19].

Consistent with previous studies we found that CHD was a common factor associated with IE in children, being present in 36% of paediatric IE cases [2,20,21,22,23]. In contrast, CHD related IE made up only 1.2% of all adult IE cases. This is likely related to the fact that as patients age, acquired valvular conditions far outnumber CHD related lesions as a potential IE substrate [24]. In children, other conditions including intravenous alimentation, longer duration of stays in intensive care units with indwelling catheters, prosthetic material, immunosuppression, or cancer therapy have also been related to an increased risk of IE, but these are less prevalent in relation to CHD in this age group [20].

### Strengths and Limitations of the Current Study

To our knowledge this study includes the largest cohort of IE cases in CHD (>2500 cases) in the literature as well as a control group of >150,000 IE cases in non-CHD patients. Capitalizing on the large sample size, the power to detect significant differences between groups is very high. As the underlying administrative dataset is complete for all hospitalizations and relevant ICD codes, the spectrum of co-morbidities (especially non-cardiac conditions) is well covered. Limitations of the study include factors directly related to the policy of anonymizing data in the registry. As no patient-specific identification codes are allocated to cases, hospitalization episodes of the same patient cannot be linked. Thus, we cannot comment on recurrent IE in the same patients. Similarly, risk factors for IE present before admission are not coded and this particularly includes dental procedures or the use of antibiotic prophylaxes in this cohort. Furthermore, we lack data on imaging findings, positive blood cultures, and the spectrum of identified bacterial organisms as well as the medical treatment regimen in this cohort. Unfortunately, we could not analyse the risk by underlying diagnostic groups. Strict anonymity rules prevent analyses where groups of patients with less than approximately six cases might be generated. As we sub-stratified IE cases by age and mortality, this would have occurred if smaller diagnostic groups would have been added to the analysis. Therefore, we decided to focus on disease complexity groups, thus avoiding this issue. Lastly, as atrial septal defects share an ICD-10 code with persistent foramen ovale in the German ICD system, patients with isolated atrial septal defects could not be included in this analysis.

## 5. Conclusions

IE accounts for a minority of CHD related hospitalizations. However, IE remains a deadly disease with mortality rate of 6% with a high proportion of cases requiring surgical intervention. MAE are common in this setting with occurrence in almost half of the IE cases. Mortality and adverse outcomes are mainly related to the demographic and co-morbidity spectrum encountered in these patients, rather than CHD complexity in the current era.

## Figures and Tables

**Table 1:** List of relevant ICD-10 or OPS codes utilized. CHD = congenital heart disease; ICD = International Classification of Diseases; OPS = Operation and Procedure key codes.

Condition	ICD-10/OPS Codes
Identification and classification of CHD	
*Complex CHD*	
Univentricular heart	Q20.1, Q20.2, Q20.4, Q22.6, Q23.4, (Q22.0 andNOT Q21.0)
Eisenmenger syndrome	I27.8 and additional Q code, Q21.88
Transposition of the great arteries	Q20.3, Q20.5, Q25.8, Q25.9
Other complex CHD lesions	Q20.0, Q26.2
*Moderate complexity CHD*	
Tetralogy of Fallot including pulmonary atresia with ventricular septal defect	Q21.3, Q21.80, (Q22.0 and Q21.0)
Ebstein anomaly	Q22.5
Aortic coarctation/interrupted aortic arch	Q25.1, Q25.2
Atrioventricular septal defect	Q21.2
Partial anomalous pulmonary venous drainage	Q26.3, Q26.4
*Simple CHD*	
Ventricular septal defect	Q21.0
Patent ductus arteriosus	Q25.0
Valvular lesions	Q22.1, Q22.2, Q22.3, Q22.4, Q22.8, Q22.9, Q23.0, Q23.1, Q23.2, Q23.3
**Endocarditis**	I33.-, I38, I39.-
**Major adverse events:**	
Death or	
Acute myocardial infarction	I21.-
Recurring myocardial infarction	I22.-
Pulmonary embolism	I26.-: O88.-
Resuscitation	8–77
Sepsis	A40.-. A41.-, B36.-
Stroke, cerebrovascular bleeding	I60–I69
Renal dialysis Intubation/Ventilation	8-853, 8-854, 8-855, 8-857 8-70, 8-71
**Operations**	OPS-5-350, OPS-5-351, OPS-5-352, OPS-5-353, OPS-5-354, OPS-5-358

**Table 2:** Association between age, sex, disease complexity and co-morbidities, and death specifically in all adult patients with infective endocarditis. IE = infective endocarditis; CHD = congenital heart disease; CI = confidence interval; OR = odds ratio. Significant variables are in bold.

All IE Cases > 18 Years of Age (with and without CHD) (Endpoint In-Hospital Mortality)	*n* = 153,776		
Parameter	OR	95% CI	*p*-Value
**Age (years)**	**1.02**	**1.02–1.02**	**<0.0001**
**Male sex**	**0.92**	**0.89–0.94**	**<0.0001**
**Simple complexity (vs. non-CHD)**	**0.74**	**0.59–0.92**	**0.008**
Moderate complexity (vs. non-CHD)	0.75	0.46–1.22	0.25
High complexity (vs. non-CHD)	0.91	0.55–1.52	0.72
**Diabetes**	**1.20**	**1.17–1.24**	**<0.0001**
**Arterial hypertension**	**0.56**	**0.55–0.58**	**<0.0001**
**Heart failure**	**1.58**	**1.53–1.62**	**<0.0001**
**Renal dysfunction**	**3.35**	**3.25–3.46**	**<0.0001**
Coronary heart disease	0.99	0.96–1.02	0.50
**Oncologic disease**	**1.66**	**1.58–1.74**	**<0.0001**
**Lung disease**	**1.10**	**1.05–1.14**	**<0.0001**

**Table 3:** Association between age, sex, disease complexity and co-morbidities, and death specifically in adult congenital heart disease patients with infective endocarditis. IE = infective endocarditis; CHD = congenital heart disease; CI = confidence interval; OR = odds ratio. Significant variables are in bold.

IE cases > 18 Yars of Age with CHD (Endpoint In-Hospital Mortality)	*n* = 1795		
Parameter	OR	95% CI	*p*-Value
**Age (years)**	**1.03**	**1.02–1.05**	**<0.0001**
Male sex	0.68	0.44–1.05	0.085
Moderate complexity (vs. simple)	0.97	0.55–1.72	0.91
High complexity (vs. simple)	1.42	0.78–2.59	0.25
Diabetes	1.04	0.59–1.84	0.89
**Arterial hypertension**	**0.51**	**0.32–0.80**	**0.003**
**Heart failure**	**1.97**	**1.33–2.93**	**0.0008**
**Renal dysfunction**	**3.93**	**2.62–5.91**	**<0.0001**
Coronary heart disease	1.42	0.88–2.31	0.15
Oncologic disease	1.77	0.74–4.21	0.20
Lung disease	0.91	0.42–1.94	0.80

**Table 4:** Association between age, sex, disease complexity and co-morbidities, and death in all paediatric patients with infective endocarditis. IE = infective endocarditis; CHD = congenital heart disease; CI = confidence interval; OR = odds ratio. Significant variables are in bold.

All IE Cases < 18 Years of Age (with and without CHD) (Endpoint In-Hospital Mortality)	*n* = 1978		
Parameter	OR	95% CI	*p*-Value
Age (years)	0.97	0.92–1.02	0.18
Male sex	1.47	0.77–2.79	0.24
Simple complexity (vs. non-CHD)	1.54	0.64–3.67	0.33
Moderate complexity (vs. non-CHD)	1.28	0.48–3.41	0.62
High complexity (vs. non-CHD)	1.59	0.7–3.6	0.27
Diabetes	2.76	0.19–40.29	0.46
Arterial hypertension	0.27	0.03–2.17	0.22
**Heart failure**	**5.76**	**3.07–10.78**	**<0.0001**
**Renal dysfunction**	**9.27**	**3.97–21.62**	**<0.0001**
**Coronary heart disease**	**24.57**	**3.35–180.07**	**0.002**
**Oncologic disease**	**10.33**	**3.25–32.85**	**<0.0001**

**Table 5:** Association between age, sex, disease complexity and co-morbidities, and death specifically in paediatric congenital heart disease patients with infective endocarditis. IE = infective endocarditis; CHD = congenital heart disease; CI = confidence interval; OR = odds ratio. Significant variables are in bold.

IE cases < 18 Years of Age with CHD (Endpoint In-Hospital Mortality)	*n* = 717		
Parameter	OR	95% CI	*p*-Value
**Age (years)**	**0.92**	**0.85–1.00**	**0.049**
Male sex	2.26	0.83–6.20	0.11
Moderate complexity (vs. simple)	0.95	0.30–3.05	0.93
High complexity (vs. simple)	1.07	0.38–2.97	0.90
Arterial hypertension	0.73	0.09–6.31	0.78
**Heart failure**	**4.32**	**1.83–10.21**	**0.0009**
**Renal dysfunction**	**8.26**	**2.19–31.15**	**0.0018**
**Coronary heart disease**	**66.47**	**5.86–753.88**	**0.0007**

**Table 6:** Association between age, sex, disease complexity and co-morbidities, and major adverse events or death in all patients with infective endocarditis. IE = infective endocarditis; CHD = congenital heart disease; MAE = major adverse events; CI = confidence interval; OR = odds ratio. Significant variables are in bold.

All IE Cases (Children and Adults, with and without CHD) (Endpoint MAE or Death)	*n* = 155,754		
Parameter	OR	95% CI	*p*-Value
**Age (years)**	**1.00**	**1.00–1.00**	**0.006**
**Male sex**	**1.12**	**1.09–1.14**	**<0.0001**
Simple complexity (vs. non-CHD)	1.04	0.93–1.15	0.51
Moderate complexity (vs. non-CHD)	0.88	0.72–1.07	0.20
High complexity (vs. non-CHD)	0.89	0.74–1.07	0.21
**Diabetes**	**1.35**	**1.32–1.38**	**<0.0001**
**Arterial hypertension**	**0.85**	**0.83–0.87**	**<0.0001**
**Heart failure**	**1.54**	**1.50–1.57**	**<0.0001**
**Renal dysfunction**	**2.95**	**2.88–3.02**	**<0.0001**
**Coronary heart disease**	**1.26**	**1.23–1.29**	**<0.0001**
**Oncologic disease**	**1.22**	**1.17–1.27**	**<0.0001**
**Lung disease**	**1.07**	**1.03–1.11**	**0.0003**

**Table 7:** Association between age, sex, disease complexity and co-morbidities, and major adverse events or death in congenital heart disease patients with infective endocarditis. IE = infective endocarditis; CHD = congenital heart disease; MAE = major adverse events; CI = confidence interval; OR = odds ratio. Significant variables are in bold.

All IE Cases with CHD (Children and Adults) (Endpoint MAE or Death)	*n* = 2512		
Parameter	OR	95% CI	*p*-Value
**Age (years)**	**0.99**	**0.99–1.00**	**0.03**
Male sex	1.13	0.94–1.37	1.20
Moderate complexity (vs. simple)	0.89	0.70–1.14	0.36
High complexity (vs. simple)	0.93	0.74–1.18	0.56
Diabetes	1.45	0.96–2.19	0.07
Arterial hypertension	1.12	0.89–1.41	0.35
**Heart failure**	**2.22**	**1.83–2.69**	**<0.0001**
**Renal dysfunction**	**4.39**	**3.39–5.69**	**<0.0001**
**Coronary artery disase**	**1.90**	**1.37–2.62**	**0.0001**
**Oncologic disease**	**2.43**	**1.06–5.59**	**0.036**
**Lung disease**	**1.75**	**1.11–2.77**	**0.017**

## Data Availability

The data used is available via the German Office of National Statistics upon registration and submission of a research proposal.
